# Rights redistribution and COVID-19 lockdown policy

**DOI:** 10.1007/s10657-022-09732-x

**Published:** 2022-04-05

**Authors:** Giampaolo Garzarelli, Lyndal Keeton, Aldo A. Sitoe

**Affiliations:** 1 Department of Social and Economic Sciences (DiSSE), Sapienza – University of Rome, Rome, Italy; 2 IPEG, SEF, University of the Witwatersrand, Johannesburg, South Africa

**Keywords:** COVID-19 pandemic, Health rights, Liberty rights, Maximin equity criterion, Public and social institutions, Public policy, Rawls, D04, D78, H11, H12, I18.

## Abstract

What is the tenet upon which the public policy of lockdown by fiat experienced during the COVID-19 pandemic is based on? The work approaches this question about the rationale of the mandatory shelter-in-place policy as an interpersonal exchange of rights, but where the exchange occurs coercively instead of voluntarily. It compares, in positive political economy terms, the normative principles of utilitarianism and Rawlsianism, and shows that lockdown by fiat is a policy that is closer to a maximin equity criterion rather than to a utilitarian one. The work moreover shows, also with the aid of a thought experiment and with factual applications, that the fiat redistribution of rights to liberty in favor of rights to health—from those least affected to those most affected by COVID-19—is, in the main, a policy choice that is to be expected under certain constraints.

## Introduction

With its more than 375 million global cases (as of end-January 2022), COVID-19, the disease from the SARS-CoV-2 virus, continues to pressure governments to take difficult measures of infection containment that are unprecedented in recent history. The main form that these measures have taken throughout the world is lockdown—a government-mandated shelter-in-place policy. Notwithstanding the quick advent and approval of multiple vaccines, vaccinations that continue to take place, and even dosing of third jabs in some countries, lockdowns still occur (e.g., Austria, Australia, New Zealand, Sri Lanka, Vietnam). While it is undeniable that medical findings have advanced along many dimensions (e.g., clinical, epidemiological, etiological, preventative) since the COVID-19 outbreak, significant uncertainty (in Knight’s well-known sense) is still afoot.^1^

In this situation where epidemiological uncertainty has translated into socioeconomic uncertainty, the recurrent Scylla and Charybdis of policy seems to boil down to a choice between saving lives versus saving output. Put differently, the policymaking trade-off is about rights to health^2^ and rights to liberty, which include rights to livelihood.^3^ The purpose of what follows is to explore the top-down redistribution of these rights when the policy choice, especially as carried out by executive decree or fiat, tilts in favor of a full lockdown.^4^ The exploration is of particular importance for democracies given the liberty sacrifice required.^5^

One often reads about the doubts surrounding the genuine health-safety necessity and democratic validity of a mass quarantine, such as a lockdown, especially in terms of benefits offsetting costs. McCloskey has gone so far as to call the policy “medieval.” At the same time, she admits that government coercion can be justified in the case of merit goods and emergencies. Both are involved and intertwined in the COVID-19 case—health and pandemic (McCloskey, 2020).

Moreover, doubts have increased since it has become common knowledge that a mass quarantine does not eradicate an epidemic, but rather limits exponential growth in contagion.^6^ In effect, it appears that what a lockdown aims to achieve is the protection of the weakest individuals of society: a pandemic does not negatively impact everyone equally, but something like COVID-19 mostly negatively impacts the feeblest (above all the elderly and those with serious comorbidity—diabetes, immunodepression, tumor, etc.).^7^ To put it in the starkest possible terms: a lockdown normatively seems to value the benefit from trying to save the life with the highest chance to be taken away above any cost to society. (From now on, we will refer to the elderly and feeble or weak categories simply as elderly and those in the least affected categories as young.)

The question then becomes one of unearthing the principle or set of principles that a lockdown is based on—even if perhaps implicitly. What cost–benefit calculus leads to such drastic policy? Why is it that less refined normative criteria are preferred to more refined ones? Why do some countries continue to opt for a lockdown? More generally: is there a logical-theoretical apparatus that can explain the normative lockdown choice, particularly by decree, from a positive vantage point?

The conventional criterion hitherto considered in connection with lockdown policy is utilitarianism (e.g., Acemoglu et al., 2021; Alvarez et al., 2021; Eichenbaum et al., 2020; Jones et al., 2021). Utilitarianism assumes that each individual, in pursuit of personal interest, balances the benefits and costs of their actions, in terms of utility, both in the present and in the future. To do so, an individual maximizes well-being (or personal welfare) through setting marginal benefits and marginal costs equal. The utilitarian extension of this criterion from the individual to society is straightforward: it is the summation of each individual’s well-being into an additive welfare function. In doing so, the sum of individuals’ marginal benefits will correspondingly equal the sum of individuals’ marginal costs. Hence, as a society is the sum of the individuals that compose it and the summed marginal benefits equal the summed marginal costs, the utilitarian condition for social welfare maximization is achieved.

This approach does not consider explicitly that individuals are distinct, that there is a separateness of persons—for instance, healthy and ill, teenagers and grandparents, skilled and unskilled are all treated the same way and not differently. Phrased in terms of the public health response to COVID-19, under utilitarianism one would value the well-being of all individuals in the same relative way: one intervenes until the marginal benefits from the addition of one type of rights are equal to the marginal costs from the subtraction of the competing type of rights irrespective of the individual characteristics of who gains or loses more rights. Utilitarianism, therefore, does not take cognizance that a disease like COVID-19 affects the elderly more severely than others.

Rather than the utilitarian, additive social welfare function, our impression is that the rationale of lockdown policy is closer in nature to Rawlsian prescriptions (Rawls, 1971, 1999). More precisely, the policy brings to mind the “maximin criterion” (Rawls, 1974), which sees more redistribution from the application of what might be called absolute equity; that is, an optimal allocation occurs through redistributing benefits to (raising the utility of) the worst-off individual (or, for Rawls, group) in society. In this type of rights reshuffling, individual characteristics matter. The criterion emanates from not being able to know about where each individual stands in society with respect to potential socioeconomic opportunities, or, in the case of a pandemic, a particular vulnerability to a virus (the so-called veil of ignorance). In doubt, the preference falls on the welfare policy that avoids the most undesirable outcome for the worst-off, where, as just recalled, any individual can be under Rawlsian assumptions.

In 2020 and 2021, this welfare policy has been lockdown in most countries. For this reason, it seems valid to consider how, in practice, a lockdown can be seen as trading off rights to liberty for rights to health.^8^ Moreover, understanding the tipping of the policy balance toward a lockdown in some countries is relevant because the more we learn from the current situation, the more informed will be future policy decisions about similar emergencies.

Our argument connects to two strands of literature. The first, and more general connection is with the growing literature on COVID-19 and pandemics. Prior to 2020, there is a notable vacuum in the political economy literature about public health, especially as it relates to contagious diseases (e.g., Leeson & Thompson, 2021).^9^ However, with the emergence of COVID-19, the situation changed. Studies have been grappling with issues relating specifically to this pandemic as well as seeing what lessons past pandemics can offer (e.g., Geloso et al., 2021).^10^ In addition, as countries (democratic and not) around the world implemented lockdowns, attention also extended to the economics exploration of the nature of this unfamiliar policy (e.g., Boettke & Powell, 2021; Coyne et al., 2021; Rachel, 2020; Scheall & Crutchfield, 2021).^11^ Others point out that the private sector has a higher-than-expected potential to internalize the negative externalities from the pandemic, inferring that a lockdown is not as necessary as governments have argued (e.g., Goolsbee & Syverson, 2021; Leeson & Rouanet, 2021).

There are two differences between our work and this first strand of literature that are worth underscoring. The first lies in our attempt to more explicitly explore the drive to engage in lockdown by fiat when most (democratic) countries initially ruled out the policy as impractical, too costly, and illiberal. The second is to try to draw concrete considerations from this drive to determine the conditions under which a country is likely to trade off liberty for health. In essence, we focus more on the process of lockdown (the possible constraints leading to the policy) than on lockdown itself (the policy).

These differences bring us to the other—and most closely related—strand of literature: contractarianism (Buchanan, 2000[1975]; Buchanan & Lomasky, 1984; Buchanan & Tullock, 1962; Rawls, 1971, 1999, 2001). Though relating in some ways also to Hayek (2013[1979]; see also, e.g., Lister, 2013 and Tomasi, 2011), our more direct link to contractarianism is through the Kantian (non-Benthamite utilitarian) nexus that exists between Rawls and Buchanan (e.g., Brennan & Kliemt, 2019; Buchanan, 1965, 1976; Kliemt, 2011).^12^ For reasons of social justice, Rawls follows the more idealistic Kant about the moral precondition of the distinction among individuals, namely in the ethical primacy of the separateness of persons. Buchanan instead follows the more pragmatic Kant who defends the priority of protecting individual spheres of autonomy with an eye to facilitating freedom of choice and natural division of labor, rendering morality an emergent property of liberty from the spontaneous interactions among diverse purposive individuals.^13^ Our stance is pragmatic as well: our concern lies in trying to explain, from a positive viewpoint, the choice of lockdown policy, not to seek or establish a normative explanation about lockdown justness (or fairness). Moreover, our stance is less static than Rawls’: similarly to Buchanan, we believe that we must do our best to be aware of the feasible normative options that we face, with the understanding that, under contractarianism, there can be social legitimacy in the choice of these options even when there is not unanimous consensus (e.g., Cowen, 2021a; Munger, 2018).

These considerations allow us to interpret more broadly Rawls’ basic intuition about the separateness of persons to more explicitly include those who differ not just in employment condition and opportunity (workers) but also, among others, in endowments, life experience, luck, and, as we shall see, even health. However, our interpretation does not rest on Rawlsian group reasoning. Moreover, it does not rest on Rawlsian redistribution by reciprocity that is owed to those who contribute effort to the total economic pie despite commanding fewer resources, namely workers. Rather, it rests on idiosyncratic individual needs. This interpretation of the separateness of persons still precludes a purely utilitarian calculation (Nozick, 1999[1974]; Schmidtz, 2011).

At the same time, our stance is also not identical to Buchanan’s. Politicians might anticipate that their constituents will blame them for allowing procedurally unpalatable events to happen (a self-interested motivation), even if the cost of preventing those outcomes is ultimately larger than the loss of life as measured by standard cost–benefit analyses. But they might themselves be innately Rawlsian in the sense of naturally believing in the political will to tackle an emergency, such as a pandemic. Individuals in an original position (such as those behind the Rawlsian veil or behind a Buchanan-Tullock constitutional stage) might agree, anticipating a situation—i.e., a policy post original position—where they (or a loved one) are denied a right to health on a utilitarian basis. The denial would seem disrespectful and require a kind of cruelty that plausibly goes against liberal democratic principles (even if the denial was genuinely the relatively better option for society from a welfare standpoint).

The point to be stressed is that whatever the reason for the presence of such political will, it may not translate sufficiently quickly into a social choice (Cepaluni et al., 2021). For example, as Rawls would moreover emphasize, individuals may underestimate the risks to themselves, to wider society or to their electoral base and actually not cooperate or, at least, not immediately cooperate. Situations of urgency and necessity require a faster policy response than that usually obtained from standard democratic decision making (even under less than unanimous agreement requirements). In these cases, an immediate, albeit coarser, response (e.g., Bolton & Farrell, 1990; Bookstaber & Langsam, 1985; Gigerenzer & Brighton, 2009; Kollman et al., 2000)—such as a central, one-size-fits-all policy—substitutes political compromise from longer, more pondered reasoning about the comparatively more refined policy. It is especially in these time-pressed cases that, holding all else constant, one would expect the protection of the elderly to trump the protection of the young by means of a “forced exchange” (Epstein, 2005) of rights to liberty for rights to health. Yet, as we also point out, a fiat lockdown response need not be expected to be necessary in the absence of other constraints (e.g., hospital capacity) and if previous experience on similar crises has coalesced into the institutional fabric, whether formal (e.g., legislation establishing standardized emergency responses to infection testing) or informal (e.g., a norm of behavior, such as voluntarily wearing protective masks in the presence of airborne diseases).

## Policy problem and policy context

Similarly to the case of the city of Wuhan, Hubei province, China, the epicenter of the COVID-19 pandemic, most countries experienced at least one lockdown. In Italy a full national lockdown, lasting more than two months, started on March 9, 2020, anticipating by two days the formal pandemic declaration by the WHO. Other European countries (e.g., France, Spain) followed suit. While in democracies in the developing world, such as India and South Africa, a similar lockdown occurred later as the virus spread across the globe.

In countries where formal governance does not allow government to interfere with the administrative sector, which can include the public health agency, the (initial) policy decision mostly leveraged on culture. This is the case of Sweden, where the belief that nudging individuals to stay at home whenever possible is sufficient to elicit a binding response.^14^ For South Korea (officially, Republic of Korea) and Taiwan (officially, Republic of China), the nature of the policy response was also not drastic, and relatively quick and experimentally multifaceted—mostly based on tracking of the infected through phone apps and swab testing at drive-through facilities. The success of the South Korean and the Taiwanese policy experiments rests on advanced technological know-how, shared values about early, broad testing, devolved public governance and, above all, previous epidemic experience with 2009’s H1N1 and 2015’s MERS outbreaks.^15^ At the same time, South Korea, Sweden and Taiwan adduced the maintenance of basic civil liberties as the fundamental core of their policy vision. (Even though South Korean and Taiwanese policy created some negative repercussions from privacy infringement.) Note that besides South Korean, Swedish and Taiwanese reasons, other ones are and were adduced to not lockdown (from not having powers to do so, as in Japan and elsewhere, all the way to denialism, as in Tajikistan at first, and even lies about the genuine nature of the emergency).

In some democracies, a full-blown general policy response was, at least in the early stages, absent. That is to say that a minimum policy of common-sense caution was in some countries the nationally mandated norm (physical distancing, reduction of large social gatherings, etc.), but that otherwise most policy decisions, such as closing of shops and other public venues, remained locally devolved. This was also the most common early response from federations, such as Canada and Switzerland. Yet ultimately of the 12 countries that have not hitherto implemented a full national lockdown, only one (Belarus) is an established autocracy.

Initially, there also was concern regarding how most African countries would fare in the face of COVID-19. However, the result was better than expected, especially for those countries that had experience in dealing with previous region-wide epidemics, such as Ebola. These countries started implementing policy (e.g., cancelling flights, introducing curfews) even before the first case of COVID-19 reached their borders. Then, once COVID-19 had entered the countries, community level interventions (testing, contact tracing, supporting individuals in home isolation) that were established under Ebola, were adapted for COVID-19 (e.g., Chua et al., 2021).^16^ Through these interventions, the number of COVID-19 infections and deaths were lower than anticipated. And yet only Burundi and Tanzania in the end did not opt to lockdown.

**Fig. 1:**
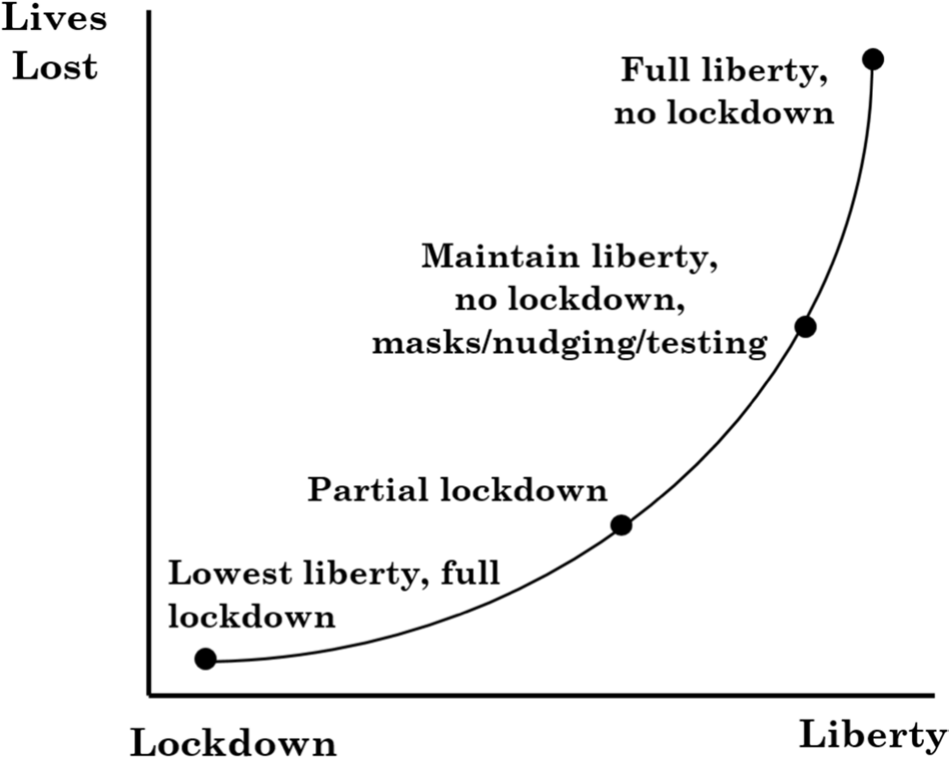
Lives lost versus liberty

Figure 1 intuitively illustrates the spectrum of the policy trade-off under a pandemic like the one that we are experiencing. At one extreme we have no lockdown, where there is pre-pandemic liberty and no policy of containment; on the other extreme we have full lockdown; and, in between, we have partial lockdown and other measures where liberty is partially surrendered (e.g., curfews, mandatory mask wearing). The takeaway from this simple figure is that in the presence of a pandemic the spread of the disease and, ultimately, deaths are correlated with the extent of liberty.

In many ways, the policy trade-off recalls the more general one about policymaking under decentralized and centralized public governance (e.g., Besley & Coate, 2003; Pennington, 2021). In this more general case, the trade-off is arguably traceable to Tocqueville’s (2012[1835–1840]) *Democracy in America* where the advantage of decentralization rests on tailoring public good supply to local needs, namely in avoiding *policy uniformity*. The supply of a uniform policy in a way restricts individual liberty because it does not allow the full satisfaction of consumer-voter preferences. In representative democracy, pondered reasoning about a decentralized versus centralized policy response is particularly valid when there is sufficient time to reach political compromise and to try out various policy design options. An ill-defined (Simon, 1973) policy problem that does not exacerbate exponentially can be usually solved by running trials on its possible policy solutions, because gaps in cognition can be overcome through gradual mistake-ridden learning from decentralized policy experimentation (Garzarelli & Keeton, 2018).

Experimentation on vaccines as a pharmaceutical policy response comes to mind. However, valid results from experimentation take time. In the case of COVID-19 many experiments were performed in parallel, and vaccines were developed and approved in record time. But production of vaccines and, especially, a vaccination campaign to reach herd immunity still take time. Meanwhile a pandemic does not stop, usually galloping at faster pace, and virus variants appear as well. One germane constraint is therefore time. Lack of time prevents an incremental, tailored response from mistake-ridden learning by distributed policy design. It prevents also long, accommodating negotiations to reach political compromise for a multipartisan policy solution. And in the immediate run both these favor a prompt—if less-refined—non-pharmaceutical response, such as coercive rights-redistribution through lockdown by executive decree.

Within our more specific context, decentralization was also the rational policy response in the face of an exogenous constraint known as the *epidemiological transition* (Omran, 2005)—a phase that many countries, both developed and developing, have been undergoing, for some time, from communicable to non-communicable diseases (e.g., cancer, diabetes, heart disease, mental illness). In the last decade or so, in fact, non-communicable diseases accounted for 70% of all global deaths (Allen, 2017). In terms of policy, this established transition put pressure on governments, especially those that protect health mainly through publicly-funded healthcare, to change priorities in healthcare service. In these cases, there is usually the concomitant that rights to health and rights to healthcare are inalienable individual rights.

Consider Italy, where the right to health is constitutional.^17^ Italian healthcare constraints in the face of the pandemic are in part also reflective of the earlier policy choice directed toward facility re-organization and spending for non-communicable diseases from the epidemiological transition. That is, they reflect a health rights policy that favors prevention rather than hospitalization. Decisions about health coverage priorities and how to spend funds earmarked for healthcare shifted to where idiosyncratic health needs are, namely sub-nationally—to the regions. Catering for non-communicable but well-identified morbidity requires the supply of ad hoc services locally because that is where the relevant knowledge about the most pressing health issues usually is. Recent data indicate that regions ultimately maintained sufficient intensive care spots, but simultaneously reduced overall hospitalization capacity.^18^ As we will see in our factual application, other countries share a policy experience that is similar to the Italian one.

In countries that have responded to the epidemiological transition, hospitals were mostly redesigned for non-contagious diseases (complex therapy, life-saving surgery, life-support, specialized diagnostic test, trauma, etc.). The implication from this technologically constrained situation from the sensible policy response to the transition is that a lockdown was seen as a political choice of self-preservation. Under a pandemic, failure of the healthcare system could be disastrous, because it would also generate negative health rights externalities for individuals needing care from non-communicable diseases; that is, hospital congestion from a pandemic impacts also those who need medical attention unrelated to the pandemic.

Therefore, a decentralized policy response toward the epidemiological transition later militated in favor of a centralized policy response toward the COVID-19 pandemic. Notice the difference: the transition can be likened to a well-defined problem where time is a soft constraint; while the pandemic to an ill-defined problem where time is a hard constraint.

At the same time, while the elderly are universally identified as vulnerable categories, after all these months matters are still unclear about some types of infected (e.g., children) and the effects on other types of categories. For example, the identification of who is contaminated and, as a result, can contaminate, is at times not straightforward as not everyone displays visible symptoms (cough, fever, tiredness) (e.g., Luo et al., 2020). Uncertainty also still exists regarding the duration of immunity after recovery from COVID-19, about the origin of the virus, and the efficacy of vaccines on new variants, such as Omicron. The pandemic is still not fully behind us.

We live in a world of constraints, and it is these constraints that often guide our decisions, including, we must not forget, policy ones. Relatedly, since we also live in a world of change (Hayek, 1948), it is important to also keep in mind that, for a variety of reasons (growth of knowledge, legislation, politics, previous policy choices, technology, etc.), constraints themselves may change, correlate, and simultaneously bind. The lesson: in policymaking, the problem faced matters as much as the idiosyncratic context.

## To trade off or not to trade off?

### Rawlsian justice

*A Theory of Justice* concerns the ethical role that ideally designed institutions can play to contribute to the common good by obviating unfavorable natural and social circumstances (Rawls, 1971, 1999). It sets forth the conditions under which free, rational and reasonable individuals choose principles of justice in society, namely those rules perceived as fair for the benefit of all in terms of distribution of basic rights and responsibilities.^19^ These conditions emerge from a thought experiment: a social contract in a hypothetical original position. In this position individuals step behind a veil of ignorance where all specific knowledge is removed from their minds. That is to say, the contractual stage in the original position is that moment where individuals are allowed general knowledge (e.g., knowledge of political and economic issues) but not idiosyncratic knowledge (e.g., knowledge of their identities and future positions in society, their capabilities, their attitudes toward risk, the economic and political status of the country to which they belong).

This epistemic situation allows impartial decision on first principles of justice because individuals stand as equals in the sense that they have the same knowledge. Moreover, since in the original position individuals do not have knowledge of the probability distribution of expected outcomes or even their current standing, there is uncertainty about one’s final standing in society. As a result, individuals will choose to safeguard the welfare of the member of society with the lowest standing, in effect, providing insurance for themselves against the risk that they may end up in that position. Since individuals stand as equals, free of any bias, agreements in the original position are fair, i.e., we have justice as fairness*.*

Within the hypothetical original position, agreement will generate two principles of justice. The first is the liberty principle, which establishes that,

[e]ach person has the same indefeasible claim to a fully adequate scheme of equal basic liberties, which scheme is compatible with the same scheme of liberties for all (Rawls, 2001, p. 42).

The liberty principle is a politically foundational—or constitutional—principle affirming that all persons in a society have the same basic rights and liberties, where the Rawlsian notion of basic liberties is a sort of “term of art”^20^ that includes both civil and political liberties, such as the right to vote and to be eligible for public office, freedom of speech and assembly, and freedom from arbitrary arrest.^21^ The liberty principle is in place to assure society’s mutually beneficial cooperation in everyday life, viz., justice is equal rights for all under normal conditions (e.g., Rawls, 1999, pp. 109–112).

According to the second principle, constituted by two parts,

[s]ocial and economic inequalities are to satisfy two conditions: first, they are to be attached to offices and positions open to all under conditions of fair equality of opportunity; and second, they are to the greatest benefit of the least-advantaged members of society (Rawls, 2001, pp. 42–43).

Rawls calls this second principle the fair equality of opportunity and the difference principle. The fair equality of opportunity component of this principle asserts that individuals with the same ambitions and talents be granted the same opportunities to education and employment no matter their background. If the first component about fair equality of opportunity is seldom caviled at, the second component has instead caused significant debate. It is in fact the difference principle that establishes that, for justice, primary goods (e.g., income, opportunities, social bases of self-respect, wealth) can be redistributed when by so doing there are benefits to the “least advantaged” or “least fortunate” (Rawls, 1971, 1999).^22^ Alternatively stated, the difference principle postulates that a society’s inequalities are fair when they are structured so as to place the worst off in their possible best position. (Thus, as others have also commented, Rawls’ theory is egalitarian but not necessarily equalizing.)

The prescriptive content of the two principles is lexicographically (“lexically”) ordered as: liberty $\succ$ fair equality $\succ$ difference. The justification Rawls offers for this ordering is as follows. The marginal benefit of basic liberties increases with income and wealth. There is a critical level of income and wealth beyond which it is irrational for an individual behind the veil to trade off liberties for income and wealth, no matter how great the increase in the latter two is. The reason is that at the critical level of income and wealth, society would have already secured primary goods—those more urgent wants such as self-respect or self-esteem. Because only less urgent wants remain to be secured, obstacles for the pursuit of equal basic liberties would have been significantly reduced. Therefore, the lexicographical ordering establishes that one would not be allowed to improve the position of the least advantaged in terms of primary goods by violating another individual’s basic liberties—justice as fairness does not permit it. In different terms, the liberty principle does not allow trade-offs between liberties and other primary goods, but within liberties trade-offs are permissible (Rawls, 1999, pp. 266, 476).^23^

Only once the liberty principle is fulfilled, the second principle takes effect. That is, the difference principle permits trade-offs among liberties and between liberties and primary goods. Additionally, under the difference principle, inequality is no longer unjust if, as we saw, the least advantaged individuals (or, for Rawls, group) in society benefit from the trade-off. Or, alternatively, inequalities are permitted if they prevent the worst possible outcome. The reason is simple. The computation of the expected value of income and wealth requires the knowledge of the probability of each possible outcome. Since the probability cannot be computed with a negligible margin of error, the rational course of action is to choose that option that minimizes the worst possible outcome: avoid outcomes that make the least advantaged individual in society worse-off.

We interpret the second principle to hold that … differences [in life-prospects] are just if and only if the greater expectations of the more advantaged, when playing a part in the working of the whole social system, improve the expectations of the least advantaged. The basic structure is just throughout when the advantages of the more fortunate promote the well-being of the least fortunate, that is, when a decrease in their advantages would make the least fortunate even worse off than they are. The basic structure is perfectly just when the prospects of the least fortunate are as great as they can be (Rawls, 1969, p. 66).

Take note that, in a strict sense, primary goods are of two types—social and natural. Social primary goods include income, liberties, opportunities, rights, and wealth; other primary goods, such as health, imagination, and intelligence, are natural (Rawls, 1999, p. 54). This distinction entails that Rawls’ structure of society does not allow the trade-off between social and natural primary goods. Rights to health cannot be traded off with, e.g., liberty in economic opportunity, even by fiat, and even if the trade-off improves the position of the disadvantaged. The reason is that social goods are about the basic structure of society, but natural goods are not. And, since in a Rawlsian society all individuals are healthy and able-bodied, one would not anticipate the need to trade off the health rights that individuals are entitled to.^24^ This clearly holds under normal circumstances where lexicographical ordering subsists (“justice as fairness”). However, under “extenuating circumstances” (Rawls, 1999, p. 55), such as a pandemic or similar crisis, matters are less clear cut, leaving open the possibility that there can be scope for trade-offs between the two types of primary goods—even, we may add, by fiat. (See also Sen, 2006, pp. 219–220.)

The discussion suggests that it is reasonable to consider the difference principle and utilitarianism as viable principles for policy alternatives. Under both the difference principle and utilitarianism liberties can be traded off with primary goods. But since both the difference principle and utilitarianism are fundamental principles of society, the attention turns to their social welfare functions, $W( {U_{i} } )$, where $U_{i}$ is an individual’s utility. The difference principle is concerned with maximizing the welfare of the least advantaged individual, which in practical terms translates into the maximin criterion: ${\text{max }}W = {\text{min}}( {U_{1} ,U_{2} , \ldots U_{n} } )$.^25^ Utilitarianism—which is at the same time, if somewhat implicitly, both a principle (what Rawls dubs the “average principle”) and a welfare criterion—is instead concerned with maximizing the welfare of the average individual: ${\text{max }}W = \frac{1}{n}\mathop \sum \nolimits_{i = 1}^{n} U_{i}$. See Table 1.

**Table 1:** A tabulation of the insights

Principle	Primary goods		Criterion
	Liberties	Others, such as health	
Liberty	Not tradable	Tradable	Maximize welfare of every individual: $\max W = \sum\nolimits_{i = 1}^{n} {U_{i} }$
Difference	Tradable	Tradable	Maximize welfare of least advantaged individual: $\max W = {\text{min}}\left( {U_{1} ,U_{2} , \ldots U_{n} } \right)$
Utilitarianism	Tradable	Tradable	Maximize welfare of average individual: $\max W = \frac{1}{n}\sum\nolimits_{i = 1}^{n} {U_{i} }$

**Fig. 2:**
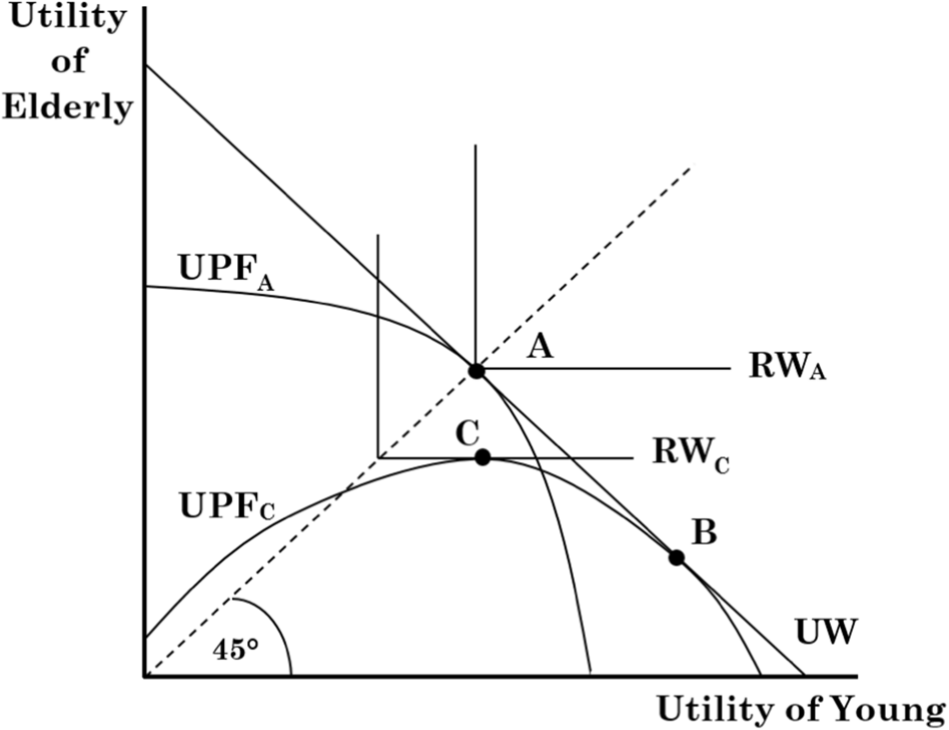
Rawlsian welfare versus utilitarian welfare

Figure 2 illustrates the potential for trade-offs between Rawlsian welfare under the maximin criterion and utilitarianism with the pandemic in mind. The initial (pre-COVID-19) utility possibility frontier for society is shown by ${\text{UPF}}_{{\text{A}}}$. The utility possibility frontier is symmetric around the 45-degree line to reflect the fair distribution of possible welfare to both young and elderly, namely those least affected and those most affected by COVID-19. Pre-COVID-19, both utilitarian welfare and Rawlsian welfare are at the same level as the welfare maximization points of both the utilitarian welfare function $\left( {{\text{UW}}} \right)$ and of the initial Rawlsian welfare $({\text{RW}}_{{\text{A}}}$) function are at the intersection of the ${\text{UPF}}_{{\text{A}}}$ along the 45-degree line (point ${\text{A}}$). Moreover, at point ${\text{A}}$, the utilities of both the young and the elderly are equal.

However, COVID-19 is an exogenous shock skewing the utility possibility frontier towards those less affected by the pandemic, namely the young, as shown by ${\text{UPF}}_{{\text{C}}}$.^26^ While trade-offs between young and elderly are possible along both utilitarian and Rawlsian lines, the utilitarian trade-off must maintain the marginal benefit and marginal cost equality: the new welfare maximizing point with utilitarian welfare is ${\text{B}}$, where ${\text{UW}} = {\text{UPF}}_{{\text{C}}}.$

To continue the utilitarian analysis of welfare, the assumption is that the total welfare for society is at the same level as pre-COVID-19 as society remains on the initial utilitarian welfare curve, ${\text{UW}}$. But, as Fig. 2 further shows, utilitarian welfare has increased for the young at the expense of the elderly. Think about how in many countries hospital congestion was overcome with a change in triage procedures: hospitals refused to care for the elderly succumbing to COVID-19 and only focused on more treatable COVID-19 cases, keeping capacity for other health conditions. This is a particularly clear illustration of a coercive change in health rights that indicates that a lockdown policy to protect the welfare of the elderly is not consistent with utilitarian welfare. Under Rawlsian welfare, however, during COVID-19 utility is maximized at point ${\text{C}}$, where ${\text{RW}}_{{\text{C}}} = {\text{UPF}}_{{\text{C}}}$. On the whole, society’s welfare has now decreased as the new maximization point is on a lower Rawlsian welfare curve $({\text{RW}}_{{\text{C}}}$ < ${\text{RW}}_{{\text{A}}} )$.

Still, at point ${\text{C}}$, the elderly have higher utility than under utilitarianism; and, notably, the utility distribution between the elderly and the young is fairer (in the Rawlsian sense) as the point is closer to the 45-degree line than under utilitarian welfare. Hence, a policy of lockdown is consistent with Rawls’ approach where some initial utility is sacrificed to ensure that the least advantaged have the highest possible utility.

The upshot, earlier hinted at, is that utilitarianism does not fit the bill. While rights, including those of liberties, can be traded off, the rights will only be traded off in such a way as to set marginal benefits equal to marginal costs, as the utilitarian welfare marginal rate of substitution remains constant. Utilitarianism, therefore, does not explicitly study issues of redistribution accounting for individual differences. Let us now consider Rawlsian welfare and the maximin criterion in greater detail.

### Trading off rights to liberty and rights to health

In the case of COVID-19, the maximin criterion is most likely going to call for the preservation of the lives of those most vulnerable to the disease by choosing health over liberty. What does this imply in terms of the possible trade-offs?

Let us remain within our society composed of two types of individuals: those who value rights to liberty more than they value rights to health (young); and those who have the opposite preference ordering (elderly). In the presence of a pandemic, trade-offs must be made between these two types vis-à-vis rights to liberty and rights to health. If the government follows a fiat policy of lockdown, then the benefits outweigh the costs for the elderly as the right to health is ranked above the right to liberty. Conversely, if the government follows a hands-off policy of no lockdown, then the benefits outweigh the costs for the young as the right to liberty is ranked above the right to health.

**Fig. 3:**
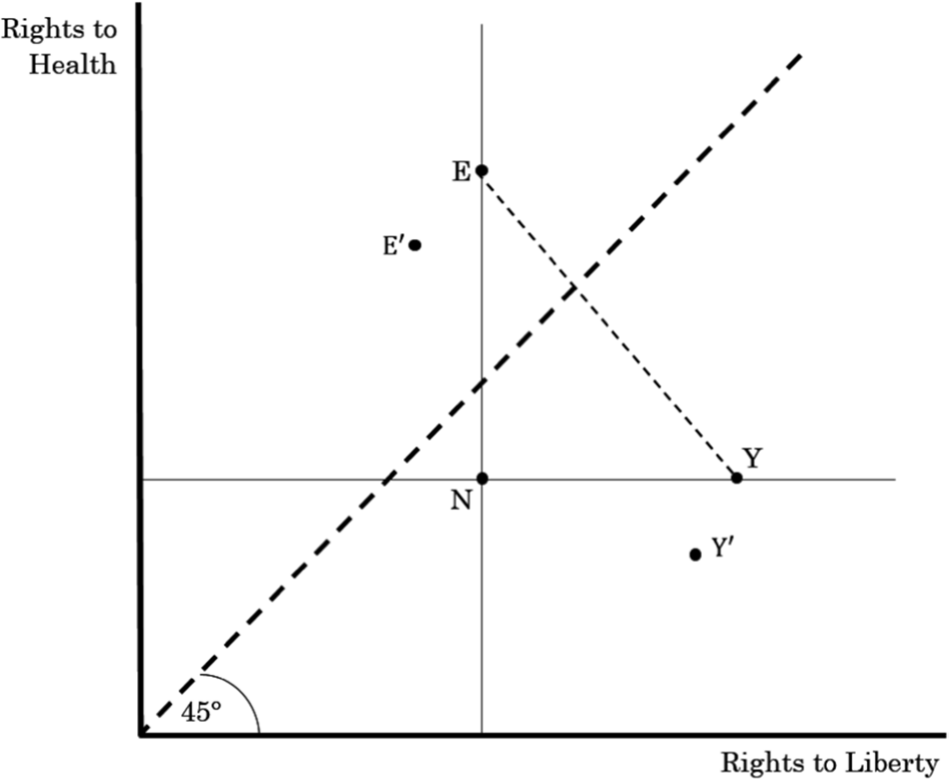
Rights trade-offs between health and liberty

Figure 3 depicts the trade-off graphically by measuring rights to health on the vertical axis and rights to liberty on the horizontal axis. Rights to health thus increase as we move upwards on the vertical axis and rights to liberty increase as we move rightwards on the horizontal axis. The 45-degree line illustrates all points of equal distribution between rights to health and rights to liberty. The points preferred by the elderly are to the left of the 45-degree line, such as ${\text{E}}$ and ${\text{E}}^{\prime }$, where rights to health exceed rights to liberty. The points preferred by the young are to the right of the 45-degree line, such as ${\text{Y}}$ and ${\text{Y}}^{\prime }$, where rights to liberty exceed rights to health. Point ${\text{N}}$ is the total welfare of the entire society in the presence of COVID-19. Any point north-east of ${\text{N}}$ is Pareto superior because at least one type of rights increases without decreasing the other. Points on segment ${\text{EY}}$, inclusive of ${\text{E}}$ and ${\text{Y}}$, represent the maximum possible total welfare absent COVID-19. (The familiar diminishing marginal rate of substitution is at work along a given, typically shaped indifference curve.)

Suppose that two policy choices are possible at point ${\text{N}}$, which is a position of partial lockdown. If government chooses a policy of no lockdown, point ${\text{Y}}^{\prime }$ is the result where rights to health fall below point ${\text{N}}$. If, instead, government chooses a policy of complete lockdown, point ${\text{E}}^{\prime }$ is the result where rights to health rise above point ${\text{N}}$. A Rawlsian policy favors society’s worst-off. So it would reject ${\text{Y}}^{\prime }$ in favor of ${\text{E}}^{\prime }$. While this would not be a Pareto improvement on welfare, a Rawlsian policy sacrifices the rights of some people to benefit others to maximize the welfare of the least advantaged in society.

To reiterate, if the trade-off is made under utilitarianism, the government will be willing to sacrifice the rights of some to benefit others, but only as far as the marginal cost of the sacrifice is less than or equal to the marginal benefit of that sacrifice. Hence, a lockdown policy choice is likely to occur in a utilitarian society only when marginal cost is less than or equal to marginal benefit. A lockdown policy is instead justified under the maximin criterion even when the marginal cost is more than marginal benefit as it is the policy that is most likely to protect the most vulnerable individual from COVID-19.

### A taxonomy from a thought experiment

In many countries, the lockdown policy was introduced without legislative process. This is not uncommon in democracies that allow for executive decrees in the presence of situations of urgency and necessity. A case in question is Italy. In a Parliamentary speech of April 30, 2020, during his second cabinet (5 September, 2019–13 February, 2021), Prime Minister Giuseppe Conte adduced *a no way out* argument by invoking Calabresi and Bobbitt’s (1978) *Tragic Choices* when justifying ex post the lockdown by executive decree. In his words,

[c]onstitutional law – and this is something that I want above all to remind to myself – … is equilibrium, equilibrium in the relationships among the powers, equilibrium of rights and guarantees. When, as in this emergency situation, the right to life and the right to health are at play, goods that besides having the character of being fundamental … , themselves constitute the prerequisite for the enjoyment of any other right, then choices, no matter how tragic, as Guido Calabresi would say, become even obligatory … .^27^

More generally﻿, a pandemic is much like being dragged into war (Pearl Harbor), being under terrorist attack (9/11) or being subject to natural disaster (L’Aquila earthquake)—situations often requiring urgent and necessary central policy responses. When considering the “emergency powers” of a “model constitution,” Hayek put it in the following terms:

[t]hough normally the individuals need be concerned only with their own concrete aims, and in pursuing them will best serve the common welfare, there may temporarily arise circumstances when the preservation of the overall order becomes the overruling common purpose, and when in consequence the spontaneous order, on a local or national scale, must for a time be converted into an organization. When an external enemy threatens, when rebellion or lawless violence has broken out, or a natural catastrophe requires quick action by whatever means can be secured, powers of compulsory organization, which normally nobody possesses, must be granted to somebody. Like an animal in flight from mortal danger society may in such situations have to suspend temporarily even vital functions on which in the long run its existence depends if it is to escape destruction (Hayek 2013[1979], pp. 458–459).

A lockdown by fiat is a manifestation of policymaking under urgency and necessity—or, if you prefer, emergency—that can be reconcilable with representative democracy if checks and balances remain intact and the centralization of executive power, as the emergency policy itself, has an explicit expiration date. Hungary under COVID-19 is in this sense the most obvious negative heuristic. (See also the classic Higgs, 1992.)

A lockdown therefore may be invoked in the presence of certain conditions and constraints. Let us elaborate this claim through a thought experiment in the manner of Rawls: the distribution of rights to health and of rights to liberty pre-lockdown can be likened to an original position of sorts behind the veil about who may or may not be infected to consider whether a policy of lockdown or no lockdown would be pursued.^28^

**Fig. 4:**
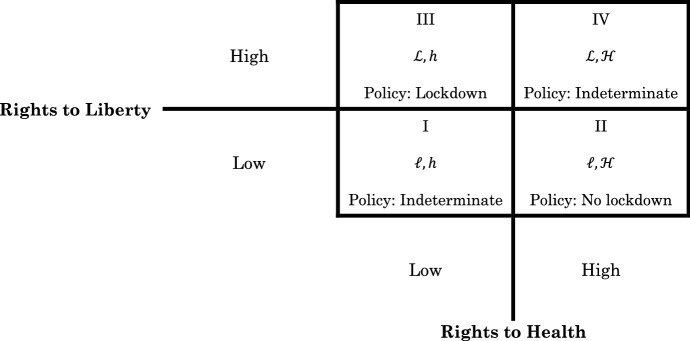
Pre-lockdown expectation of policy choice. Note: ${\mathcal{L}} > \ell$ and ${\mathcal{H}} > h$

We can imagine four possible positions of pre-lockdown rights distribution—i.e., before the COVID-19 pandemic. Consider Fig. 4. In each box, the left-hand entry indicates the “level” of rights to liberty $\left\{ {{\mathcal{L}},\ell \left| {\mathcal{L}} \right. > \ell} \right\}$ and the right-hand entry indicates the “level” of rights to health $\left\{ {{\mathcal{H}},h\left| {\mathcal{H}} \right.> h} \right\}$.

In Box II rights to health are high but rights to liberty are not $\left( {\ell ,{\mathcal{H}}} \right)$. Hence, it is unlikely that rights on liberty will be given up to gain additional rights on health. Instead, in this case, it is more likely to have a policy that gives up the rights that are at a high level (rights to health) for the rights that are at a low level (rights to liberty). In Box III, rights to liberty are high but rights to health are not $\left( {{\mathcal{L}},h} \right)$. Here it is more likely that a policy gives up rights to liberty in exchange for rights to health as there is a sufficiently high level of liberty to concede the trade-off. (We again have the diminishing marginal rate of substitution at work.)

In Box IV both rights to liberty and rights to health are at high levels $\left( {{\mathcal{L}},{\mathcal{H}}} \right)$. It would seem therefore that whether more importance is granted to rights to liberty or to rights to health cannot be determined a priori. (See also Cooter, 1989.) This is the conclusion reached in Box I, too. But Box I originates from a less favorable position as both rights to liberty and rights to health are low $\left( {\ell ,h} \right)$. Thus, one cannot establish what type of rights would be traded off behind a veil in Box I and Box IV. In practice, this means that in countries where there is no clear willingness or unwillingness to trade off liberty rights for health rights (or vice versa) and that have locked down, the lockdown motivation(s) may lie elsewhere. (This is a matter that we will return to shortly.)

The four possible positions of rights distribution pre-lockdown policy from our thought experiment suggest that only countries with rights distribution pre-lockdown akin to those in Box II would not implement a complete lockdown policy. That is, behind a veil of ignorance, Box II countries would not trade rights to liberty in exchange for rights to health. Conversely, a country with rights distribution pre-lockdown akin to those in Box III would pursue a policy of complete lockdown: behind the veil, Box III countries would consider justified a trade of rights to liberty in exchange for rights to health.

We now push the thought experiment further to attempt to determine whether a lockdown by fiat will occur within democratic countries. To do so, we need to perform two tasks. First, we try to identify factual equivalents to the four boxes from the thought experiment, that is, which countries fit each of the four boxes in Fig. 4. Second, we lift the veil to solve for the indeterminacy in boxes I and IV.

## Seeking concreteness, behind the veil

### Data

Still in keeping with Rawls, we consider only democracies, which narrows our sample down to 101 countries (listed in Table 5 of “Appendix 1”). We use the Polity2 index from Polity 5 to identify a country’s political regime.^29^ The Polity2 index has a minimum value of − 10 and a maximum value of 10, with a higher value indicating fuller democracy. Other political regimes include autocracies (countries with a Polity2 value between − 10 and − 6), closed anocracies (countries with a Polity2 value between − 5 and 0), and open anocracies (countries with a Polity2 value between 1 and 5). All countries with a Polity2 value between 6 and 10 are democracies. Thus, they are included in our sample.

To measure rights to liberty we use three indices jointly compiled by the Cato Institute and the Fraser Institute, namely personal freedom, economic freedom, and human freedom.^30^ All three measures of liberty have a minimum value of 0 and a maximum value of 10, with a higher value indicating greater liberty. All three measures cover the countries in our sample.

In Rawlsian spirit, our main measure of liberty is the Personal Freedom Index. This index originates from 34 indicators of civil and political liberties in the areas of rule of law; security and safety; movement; religion; association, assembly, and civil society; expression and information; and identity and relationships.

To measure rights to health we use the World Development Indicators (WDI) from the World Bank.^31^ The WDI cover 264 countries and contain data on 21 topics, including health. While there are over 200 indicators on health, we consider the Universal Health Coverage (UHC) Service Coverage Index as most relevant in the context of COVID-19. The UHC Service Coverage Index is the most comprehensive indicator on health as it captures various health interventions, including those in reproductive, maternal, newborn and children’s health, as well as infectious diseases, non-communicable diseases, and both access to and capacity of healthcare services.

Then, in the next section, to measure access to healthcare we continue to use the WDI, namely Hospital Beds (per 1000 people), Physicians (per 1000 people), Current Health Expenditure per Capita (US$), Average Share of Non-communicable Diseases Death in Total Deaths, and Percentage of the Population Aged 65 Years or Above. All health indicators have a minimum value of 0 and a maximum value of 100. Hospital Beds (per 1000 people), Physicians (per 1000 people) and Current Health Expenditure per Capita (US$) provide a measure of capacity of healthcare services originating from capital stock (supply side) whereas Average Share of Non-communicable Diseases Death in Total Deaths and Percentage of the Population Aged 65 Years or Above measures capacity to healthcare services originating from policy choice (demand side).

### Rights to liberty and rights to health before COVID-19

To understand the position of countries before COVID-19, we start by using the Personal Freedom Index to proxy for rights to liberty. (“Appendix 2” takes an additional step by incorporating other proxies for liberty, namely Economic Freedom and Human Freedom. However, the results remain unchanged.) We use the UHC Service Coverage Index to proxy for rights to health. Since the most recent UHC data are from 2017, we exclude countries that are not in both indicators.

**Fig. 5:**
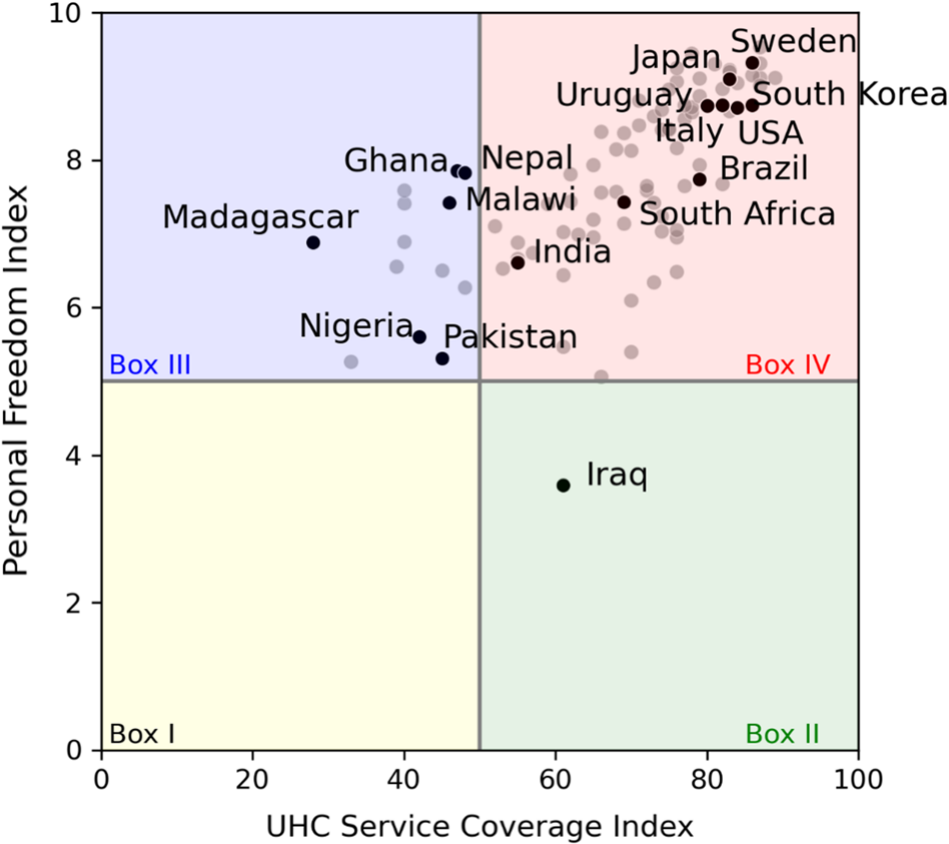
Rights distribution in democracies before COVID-19

Figure 5 provides the rights distribution between liberty and health in countries in 2017, i.e., before COVID-19, that corresponds to our theoretical expectations contained in Fig. 4. Refer to Table 5 for the complete list of countries in each box.

There are no countries in Box I, i.e., there are no democratic countries with low rights to liberty (below or equal to 5) and low rights to health (below or equal to 50). Box II contains one of the 101 countries, namely Iraq, in which rights to liberty are low (below or equal to 5) and rights to health are high (above 50). Our expectation is that Iraq would not pursue lockdown policy.

Box III shows those countries for which rights to liberty are high (above 5) and rights to health are low (below or equal to 50), which are 13 out of 101. Among others, we see Ghana, Madagascar, Malawi, Nepal, Nigeria and Pakistan. We expect that these countries would lockdown.

Lastly, Box IV encompasses 87 out of 101 of the sample countries. These are the relatively more democratic countries, that is, those exhibiting both high rights to liberty (above 5) and high rights to health (above 50). They include, among others, Brazil, India, Italy, Japan, South Africa, South Korea, Sweden, Uruguay, and the USA. As we cannot directly determine whether countries in Box IV would be willing to trade rights to liberty for rights to health, we require more information to determine whether those countries would lockdown. This requirement is taken up in the next section.

## Seeking concreteness, lifting the veil

As mentioned, neither our theoretical expectation nor our initial examination of the data can determine whether countries in Box IV will lockdown behind the veil. Let us lift the veil to consider other possible lockdown motivations, with special consideration for Box IV.

### Access to healthcare

For countries in Box IV that are indeterminate from having both high rights to health and high rights to liberty, another possibility that may motivate lockdown is insufficient access to healthcare.^32^ Thus, instead of rights to health, we now home in on rights to access to healthcare for which we use healthcare capacity as proxy. Table 2 reports three indices that measure healthcare capacity for our sample: Hospital Beds (per 1000 people), Physicians (per 1000 people), and Current Health Expenditure per Capita (US$). We learn from Table 2 that countries in Box IV have, on average, greater access to healthcare than countries in the other boxes: Box IV countries are likely to have sufficient access to healthcare and, thus, this is not likely a reason to lockdown, meaning that our expectation of indeterminacy remains unchanged vis-à-vis Fig. 4.

**Table 2:** Rights to Health: Healthcare capacity

Position	Observations	Average	Std Dev	Minimum	Maximum
Hospital Beds (per 1000 people)^a^					
Box I	0	–	–	–	–
Box II	1	1.30	–	1.30	1.30
Box III	4	0.95	0.29	0.60	1.30
Box IV	69	3.74	2.00	0.60	8.20
Physicians (per 1000 people)^b^					
Box I	0	–	–	–	–
Box II	1	0.64	–	0.64	0.64
Box III	11	0.15	0.24	0.02	0.85
Box IV	73	2.31	1.28	0.06	6.05
Current Health Expenditure per Capita (US$)^c^					
Box I	0	–	–	–	–
Box II	1	152.64	–	152.64	152.64
Box III	13	48.98	13.58	16.36	86.31
Box IV	86	1777.78	2196.36	56.54	9869.74

*Source of data*: World Development Indicators

^a^Based on 74 observations from 2011 data

^b^Based on 85 observations from 2010 data

^c^Based on 100 observations from 2016 data

### Epidemiological transition

As we saw, many countries have undergone an epidemiological transition. As a result, these countries may be willing to opt for lockdown with the understanding that their healthcare systems are unprepared for a pandemic from an infectious disease.

**Fig. 6:**
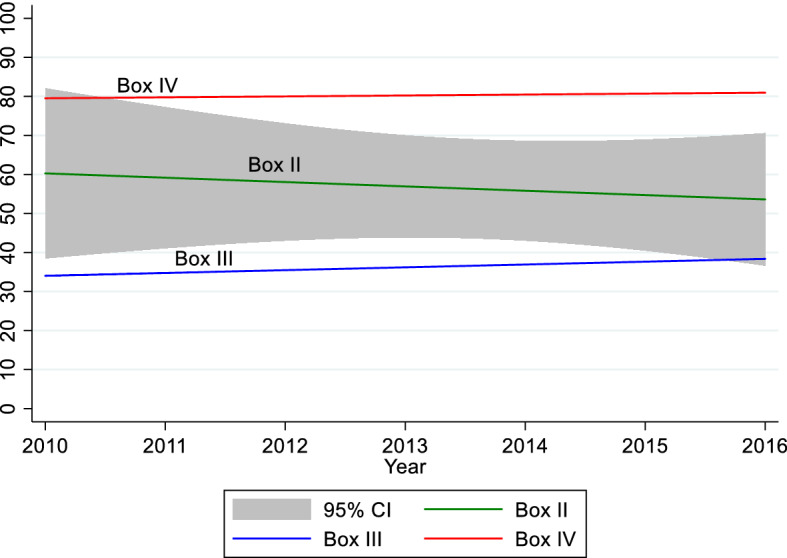
Average share of non-communicable diseases death in total deaths (2010–2016). *Source of data*: World Development Indicators

To proxy for the epidemiological transition, Fig. 6 shows the Average Share of Non-communicable Diseases Death in Total Deaths between 2010 and 2016 in our sample countries. Since at least 2010, the Average Share of Non-communicable Diseases in Total Deaths has averaged above 50% for countries in boxes II and IV and below 40% for countries in Box III. However, there might not have been a significant difference in the Average Share of Non-communicable Diseases in Total Deaths between countries in boxes II and III by 2016 since there is an overlap of confidence intervals of Average Share of Non-communicable Diseases in Total Deaths between countries in boxes II and III in 2016.

Figure 6 further suggests that the Average Share of Non-communicable Diseases in Total Deaths is highest in Box IV, and that by 2016 the average is statistically the same in boxes II and III. This entails that we have a reason for countries in Box IV to lockdown: having undergone an epidemiological transition, there is an arguably inadequate preparedness for an infectious disease pandemic. This is a piece that helps to solve the indeterminacy puzzle of Box IV: even though Box IV countries have relatively greater access to healthcare (Table 2), the type of healthcare offered tips in favor of non-communicable diseases. The consequence is that Box IV countries will most likely implement a lockdown to prepare or to convert healthcare facilities to pandemic needs.

### Median voter

Consider now a possible median voter motivation for lockdown. A representative government that is mindful of the population would likely protect the median voter, especially if the median voter is elderly and more susceptible to COVID-19. We consider how our sample fares in terms of median voter considerations in Table 3, which ranks the average share of the elderly in the total population. The largest share is in Box IV, with boxes II and III holding very close average shares of the elderly in the total population. This means that governments in Box IV countries would likely lockdown. We thus obtain a second possible reason for lockdown in Box IV countries.

**Table 3:** Percentage of the population aged 65 years or above in 2017

Position	Observations	Average	Std Dev	Minimum	Maximum
Box I	0	–	–	–	–
Box II	1	3.23	–	3.23	3.23
Box III	13	3.35	0.98	2.41	5.66
Box IV	87	12.19	6.14	2.08	27.11

*Source of data*: World Development Indicators

### Affordability of lockdown

Lastly, a possible motivation for a country to accept a lockdown is simply that it can afford to. Considering Table 4, we see that countries in Box IV tend to be wealthier than countries in other boxes: countries in Box IV have on average a real GDP per capita of US$21,866.83, which is about 4 times the average real GDP of countries in Box II and about 22 times the average real GDP of countries in Box III. In response to a pandemic, it would seem that poorer countries would not be able to afford drastic measures as much as richer countries (e.g., Barnett-Howell & Mobarak, 2020). Hence, we would expect Box IV countries to lockdown, simply since they can afford to.

**Table 4:** Real GDP per capita in 2017 (US$)

Position	Observations	Average	Std Dev	Minimum	Maximum
Box I	0	–	–	–	–
Box II	1	5637.91	–	5637.91	5637.91
Box III	13	998.19	610.36	370.75	2412.37
Box IV	87	21,866.83	22,595.27	1070.37	109,453.00

*Source of data*: World Development Indicators

At the same time, as we shall see in more detail momentarily, this lockdown expectation cannot be taken for granted. Indeed, we may obtain the opposite result of no lockdown. For richer countries tend to be institutionally more constrained than poorer ones, especially when it comes to challenging personal liberties (e.g., Troesken, 2015).

### Expectations and data

Our factual reflections are suggestive of some expectations corresponding to the boxes in Fig. 4 (and Table 5). In boxes with well-defined expectations (II and III), there is only one country in which no lockdown is the expected policy choice—Iraq in Box II. Despite this expectation, Iraq entered a lockdown. The countries in Box III are in the main the less developed ones from Africa and South Asia. Lockdown is expected in these countries as there is a will to trade the relatively higher rights to liberty with the relatively lower rights to health. In fact, as expected, these countries locked down.

In Box IV, there are 87 countries where rights to liberty and rights to health are both high. Behind the veil, our expectation for countries in Box IV is that the policy choice of lockdown by fiat is indeterminate. To solve this indeterminacy, we lift the veil to consider other reasons for lockdown, namely insufficient access to healthcare, countries having undergone an epidemiological transition, an elderly median voter, and whether or not a country can afford it. Together, these reasons point to the likelihood of lockdown in countries in Box IV. As mentioned, countries in Box IV have a greater share of elderly in their populations and higher Non-communicable Diseases Deaths in Total Deaths. Therefore, these countries face more constraints from the epidemiological transition, i.e., pre-COVID-19 public policy shifted priorities in healthcare services from communicable to non-communicable diseases. Furthermore, a higher GDP in countries in Box IV means that these countries are relatively more likely to be able to afford the lockdown.

The expectation that Box IV countries will lockdown is consistent with 80 of 87 countries that implemented lockdown. However, we see that seven countries in Box IV (Brazil, Iceland, Japan, Nicaragua, Sweden, Uruguay, and the USA) opted not to lockdown. Why? Starting with Brazil, denialism on the part of President Jair Bolsonaro led to a lack of national lockdown. Nevertheless, most federated states went against national policy, and only two of the 16 Brazilian states ultimately opted not to lockdown.^33^ Iceland, Nicaragua, and Uruguay implemented contact-tracing and extensive testing policies instead of locking down.^34^ Japan, Sweden, and the USA have institutional constraints that limit the central government’s power to lockdown.^35^ In the case of the USA, though, 44 of the 50 federated states still locked down.

## Final remarks

We attempt to identify a rationale that can help to explain the coercive reshuffling of individual rights engendered in democracies during the COVID-19 pandemic through lockdown—a blunt policy instrument that sacrifices the liberty of all to try to better protect the more delicate health of some. In fact, only 12 countries worldwide have not completely locked down.

Our contractarian analysis compares, from a positive political economy perspective, the normative principles of utilitarianism and Rawlsianism in relation to lockdown. Utilitarianism translates into an average welfare policy criterion that does not account for differences among persons: it allows trade-offs between rights to liberty and rights to health only until the marginal benefit of protecting health equals the marginal cost of restricting liberty. As a consequence, utilitarianism cannot account for lockdown policy. Rawlsian maximin, instead, is a criterion that, by protecting the weakest, concerns the separateness of persons, allowing the exchange of rights to liberty for rights to health even when the marginal benefit is outweighed by the marginal cost. Thus, mutatis mutandis, maximin equity underpins the non-voluntary rights-redistributing policy of lockdown. Through some factual considerations we additionally point out, again without passing judgement, that the fiat reshuffling of rights to liberty in favor of rights to health from those potentially least affected to those potentially most affected by COVID-19 is, in the main, a policy choice that is to be expected under certain constraints.

When the pandemic will be behind us and massive amounts of reliable data will be readily available, we will be able to more precisely grasp the full socioeconomic costs and benefits of different COVID-19 policy responses in terms of: foregone profit opportunities; debt burdens transferred inter-generationally; erosion of the tax base; loss of civil liberties or individual rights; psychological costs in terms of mere supermarket queues, loss of self-confidence, mental depression, unemployment; and the like. In other words, our sense is that we do not yet have sufficient data to crisply consider different welfare effects of different types of pandemic policy. As always, time will better inform us about the effects of different policy decisions. Still, the hope is that lessons from this experience can help to prepare most of the world for an institutional readiness that decreases coercive non-pharmaceutical discretionary interventions.

We concede that it is difficult to swiftly solve a problem that is ill-defined and mutates at a fast pace, such as a new virus, even when expected. The World Health Organization and many countries’ centers for disease control that exist for this reason are testament to this (as are Bill Gates’ advance notices about future epidemic threats^36^). Such a challenging situation combined with institutional, technological, time and arguably other constraints leads us to conclude that, as in the Buchanan-Rawls nexus, Kant still represents a point of convergence. But we reach Kant and his imperative because we show how the policymaker is challenged by the policy problem itself as well as by its embeddedness. Of course, this is different from claiming that a lockdown is just or fair.

## Appendix 1

See Table 5.

**Table 5:** List of countries

Box I	Box II	Box III	Box IV
–	Iraq	Benin, Burkina Faso, Central African Republic, Ghana, Guinea-Bissau, Lesotho, Madagascar, Malawi, Nepal, Nigeria, Pakistan, Senegal, Sierra Leone	Albania, Argentina, Australia, Austria, The Bahamas, Barbados, Belgium, Belize, Bolivia, Bosnia and Herzegovina, Botswana, Brazil*, Brunei Darussalam, Bulgaria, Cabo Verde, Canada, Chile, Colombia, Costa Rica, Croatia, Cyprus, Czech Republic, Denmark, Dominican Republic, El Salvador, Estonia, Finland, France, Georgia, Germany, Greece, Guatemala, Guyana, Honduras, Hong Kong, Hungary, Iceland*, India, Indonesia, Ireland, Israel, Italy, Jamaica, Japan*, Kenya, Kyrgyz Republic, Latvia, Lebanon, Lithuania, Luxembourg, Malta, Mauritius, Mexico, Moldova, Mongolia, Montenegro, Myanmar, Namibia, Netherlands, New Zealand, Nicaragua*, North Macedonia, Norway, Panama, Paraguay, Peru, Philippines, Poland, Portugal, Romania, Serbia, Seychelles, Slovak Republic, Slovenia, South Africa, South Korea*, Spain, Sri Lanka, Sweden*, Switzerland, Timor-Leste, Trinidad and Tobago, Tunisia, United Kingdom, United States of America*, Uruguay*, Zambia

The * refers to countries that did not lockdown. Other countries that did not lock down are: Belarus, which, being an autocracy, is out of the sample; Burundi and Tanzania which, being anocracies, are also out of the sample; and Taiwan, for which we lack data

## Appendix 2

In addition to the Personal Freedom Index, for robustness we can measure rights to liberty with the Economic Freedom Index and the Human Freedom Index, which are also jointly compiled by the Cato Institute and the Fraser Institute.^37^ All three indices cover our sample countries and have a minimum value of 0 and a maximum value of 10, with a higher value indicating greater liberty. The Economic Freedom Index is based on 42 indicators of economic liberty in the areas of size of government, legal system and property rights, sound money, freedom to trade internationally, and regulation. The Human Freedom Index is considered the most comprehensive index on liberty. The index combines 76 indicators from both personal freedom and economic freedom.

In Table 6—which is consistent with the vertical dimension of Fig. 5—we see that, on average, countries in Box II have lower rights to liberty than countries in boxes III and IV under all three indices.^38^ This result is congruous with using the measure of personal freedom only. Consequently, it does not shed more light on whether countries in Box IV would lockdown.

**Table 6:** Rights to liberty: personal freedom, economic freedom, and human freedom in 2017

Position	Observations	Average	Std Dev	Minimum	Maximum
Personal Freedom Index					
Box I	0	–	–	–	–
Box II	1	3.59	–	3.59	3.59
Box III	13	6.72	0.91	5.27	7.85
Box IV	87	7.97	1.06	5.06	9.53
Economic Freedom Index					
Box I	0	–	–	–	–
Box II	1	5.57	–	5.57	5.57
Box III	13	6.00	0.44	5.18	6.65
Box IV	87	7.38	0.71	3.61	8.91
Human Freedom Index					
Box I	0	–	–	–	–
Box II	1	4.58	–	4.58	4.58
Box III	13	6.37	0.56	5.25	7.18
Box IV	87	7.68	0.80	4.54	8.93

*Source of data*: Cato Institute and Fraser Institute

## Footnotes

1 On COVID-19 uncertainty see, among others, Baker et al. (2020) and Fauci et al. (2020). The simplest encapsulation we came across, though already slightly dated, about the uncertainty associated with COVID-19 is an Internet meme. Translating from Italian, the meme states that we are facing, echoing the thought experiment of Schrödinger’s Cat where a cat may at once be dead *and* alive, *Schrödinger’s Virus*. “Not all of us can undergo the test. Thus, we do not know whether we have the virus or not. We must behave as if we have it, so that we do not infect others. But we must behave also as if we are not yet contaminated, because it would mean that we are immune. We therefore contemporaneously have and do not have the virus. Schrödinger’s Virus” (Credit attributed to Mat Krahn).

2 By right to health we have in mind the definition found in the Constitution of the World Health Organization (WHO), namely that health is “a state of complete physical, mental and social well-being and not merely the absence of disease or infirmity,” and that the right to the “enjoyment of the highest attainable standard of health is one of the fundamental rights of every human being” (WHO, 2006, p. 1). This right was similarly expressed several decades earlier in Article 12 of the International Covenant on Economic, Social, and Cultural Rights (UN Committee on Economic, Social, and Cultural Rights, 1966). It is related to the right to access sufficient healthcare but covers broader measures (to name a few: access to clean water, sanitation, workplace health and safety). In ensuring the right to health, a noteworthy responsibility of the WHO is “to stimulate and advance work to eradicate epidemic, endemic and other diseases” (WHO, 2006, p. 2).

3 More generally, for our purposes rights to liberty include the right to consume, exchange, own, produce, and freely contract as well as the rights of economic initiative, including profit-seeking and entrepreneurship (e.g., Wu & Davis, 2004, pp. 163–164).

4 Data compiled by Wikipedia indicate that 12 countries did not lockdown—a figure that includes Brazil, where two federated states did not lockdown, and the United States of America, where six federated states did not lockdown, https://en.wikipedia.org/wiki/COVID-19_lockdowns#Countries_and_territories_without_lockdowns (last accessed on December 6, 2021).

5 Besides decision processes, what also differs according to nature of political regime (democratic or not) is the enforcement of the lockdown. During the so-called Great Lockdown in Italy (9 March, 2020–18 May, 2020), for example, a self-declaration justifing movement (for a valid reason, such as groceries, medicine, helping an elderly family member, work) was sufficient. In authoritarian China, the main entrance of some apartment buildings in Wuhan was soldered shut by government so that tenants could not freely walk out. See “Coronavirus: How the Deadly Epidemic Sparked a Global Emergency,” Four Corners Documentary, https://www.youtube.com/watch?v=ycrqXJYf1SU (last accessed on March 7, 2020).

6 Note that while it might be obvious, it is not a trivial point that a lockdown policy is not effective against non-contagious diseases. The extent of non-contagious or lifestyle related diseases such as cancer, heart disease, obesity, diabetes, mental illness, etc., are associated with how people live, vis-à-vis diet, exercise, smoking, alcohol consumption, and weight, rather than how much they interact with each other. The spread of a contagious disease, though, is directly related to how much people interact with each other (think of the basic reproductive number, ${\mathrm{R}}_{0}$). In fact, a lockdown policy may actually increase non-contagious disease by restricting exercise time, by isolating people from their friends and families, and by driving unhealthy diets and habits (e.g., Füzéki et al., 2020).

7 Compare, for instance, Roes (2018) on the Spanish Flu of 1918 and Ahmed et al. (2020).

8 Note that while rights to health and rights to access to healthcare differ, the two, as we will see, can relate in practice.

9 See also the various special issues on “The Political Economy of Pandemics” of the *Journal of Political Institutions and Political Economy*.

10 In economics, the CEPR working paper series on *Covid Economics, Vetted and Real-Time Papers*, launched at the end of March 2020, became an important reference point; even if it closed submissions on June 23, 2021, issues are still freely available online at: https://cepr.org/content/covid-economics-vetted-and-real-time-papers-0.

11 Much like the fast growing area of research on lockdown policies, related policies of social distancing (e.g., Greenstone & Nigam, 2020) and designations of essential goods (e.g., Storr et al., 2021; Thunström et al., 2020) are also under analysis.

12 In a strict sense, of course, a contractarian original position is nothing more than a type of state of nature, which we find in earlier social (and social contract) scientists and philosophers too—e.g., Hobbes, Locke, Rousseau, and even Kant. On the latter, Rawls (1999, p. 118, n. 11) writes that the original position of the veil of ignorance “is implicit … in Kant’s doctrine of the categorical imperative, both in the way this procedural criterion is defined and the use Kant makes of it. Thus when Kant tells us to test our maxim by considering what would be the case were it a universal law of nature, he must suppose that we do not know our place within this imagined system of nature.” Before Buchanan and Tullock (1962), other economists (Harsanyi, 1953, 1955; Vickrey, 1945, 1960) also made an original position assumption, but, as Rawls (1999) also points out, to make more traditional utilitarian arguments.

13 See Surprenant (2018) on Kant-the-idealistic from moral philosophy versus Kant-the-pragmatic from practical philosophy.

14 https://www.cnbc.com/2020/03/30/sweden-coronavirus-approach-is-very-different-from-the-rest-of-europe.html (last accessed on April 1, 2020) and https://www.nytimes.com/2020/03/28/world/europe/sweden-coronavirus.html (last accessed on April 4, 2020).

15 https://www.scmp.com/week-asia/health-environment/article/3074469/coronavirus-south-korea-cuts-infection-rate-without (last accessed on April 1, 2020) and https://time.com/collection/finding-hope-coronavirus-pandemic/5820596/taiwan-coronavirus-lessons/ (last accessed on December 24, 2021).

16 See also https://www.ft.com/content/c0badd91-a395-4644-a734-316e71d60bf7 (last accessed on March 15, 2021) and https://time.com/5919241/africa-covid-19-outbreak/ (last accessed on March 15, 2021).

17 See Article 32 of the Italian Constitution, available in official English translation at https://www.senato.it/documenti/repository/istituzione/costituzione_inglese.pdf (last accessed on January 9, 2021).

18 See Angelici et al. (2020). One estimate reports that before COVID-19 Italy could rely on 5,324 intensive care hospital spots, and 2,974 spots in infectious disease hospital wards. These are small numbers if one considers a population of more than 60 million, with a very high share of elderly people—23 per cent of Italians are aged 65 and over (2nd oldest population after Japan) with a median age of 45.5 (3rd highest after Japan and Germany)—and the 2,668,266 infected cases, with 92,338 fatalities, reported for Italy on February 11, 2021 on COVID-19 Tracker. (The target, slowly being achieved, is to increase the total intensive care hospital spots by 50%.) https://www.corriere.it/cronache/20_marzo_16/coronavirus-quanti-posti-terapia-intensiva-ci-sono-italia-quanti-ne-arriveranno-0fbafa76-678a-11ea-93a4-da8ab3a8afb1.shtml (last accessed April 2, 2020).

19 In Rawls’ institutional construct, individuals, especially thanks to reasonableness, understand the value of compromise. For they not only possess common sense but also a sort of common intuition about a shared destiny from their actions that aligns their incentives. See especially Rawls (1971, pp. 102–103) and, in milder form, Rawls (1999, pp. 87–88).

20 We are grateful to an anonymous referee for suggesting this expression.

21 It should now be clear that our notion of basic liberties includes economic liberties, such as freedom of enterprise and of transacting (see footnote 3), in addition to Rawls’ original civil and political liberties. Rawls (e.g., 1971, 1999) argues that social structures are dependent upon individuals’ actions. As such, the attainment and maintenance of a just basic structure requires the presence of a sense of justice among individuals in society. By allowing individuals to view each other as equals in a mutually beneficial relation, economic liberties bring out a sense of justice thereby improving upon individuals’ civil and political liberties. In brief, economic liberties can aid the formation of social cooperation, which is at the basis of Rawls’ theory of justice (e.g., Cowen. 2021b). See also the classic Buchanan and Lomasky (1984) as well as Tomasi (2011).

22 Of course, the “least advantaged” or “least fortunate” can mean different things in different contexts. As was specified at the outset, Rawls has in mind mainly the group of workers to which reciprocity is normatively prescribed for reasons of contribution to economic output and not out of need. “The least advantaged are not, if all goes well, the unfortunate and unlucky—objects of our charity and compassion, much less our pity—but those to whom reciprocity is owed as a matter of political justice among those who are free and equal citizens along with everyone else. Although they control fewer resources, they are doing their full share on terms recognized by all as mutually advantageous and consistent with everyone’s self-respect” (Rawls, 2001, p. 139). But, as was also specified at the outset, in our COVID-19 case the “least advantaged” are mostly elderly and feeble or weak individuals, to whom we refer to as the “elderly” for mere reasons of compactness.

23 Note that Rawls’ lexicographical ordering is not always consistent in terms of liberty as such being universally primary. Rawls later clarifies that he rather seems to have in mind the primacy of liberties (as also reflected in a rewording of the liberty principle over the years), but in no particular ordering (e.g., Rawls, 1996, pp. 291–292). See Lomasky (2005) for a critical take about the implications of this change, and related ones, in Rawls’ thought. Compare also the collection edited by Freeman (2003, *passim*).

24 The assumption that all individuals are healthy and able-bodied is another well-known issue with Rawls’ notion of the least advantaged. As Nozick (1999[1974], p. 190) observes, “why exclude the group of depressives or alcoholics or the representative paraplegic?” The answer would seem to be that for Rawls the exclusion simplifies the construction of his theory of justice (e.g., Arrow 1973, p. 254). Entering the issue in more detail is beyond our positive objective, but see, among others, Meadowcroft (2011).

25 This does not mean that the Rawlsian framework is about a social welfare function, but that there can be heuristic value in it being proxied by one. In a strict sense, in fact, the difference principle and the maximin criterion differ. The difference principle, being a fundamental rule about the basic structure of society, ethically establishes the separateness of persons. The maximin criterion is instead a rule for choice under uncertainty; it is, for lack of a better term, the operational level of the difference principle (Rawls, 1999, pp. 72–73). At the same time, the difference principle and the maximin criterion can also correlate, for example in the presence of very high-risk aversion (e.g., Buchanan & Faith, 1980).

26 We consider, for simplicity, that total utility remains constant, *ceteris paribus*. The absence of this consideration does not affect the logic of our reasoning.

27 “XVIII Legislatura, Resoconto dell’Assemblea, Seduta N. 333 di Giovedì 30 aprile 2020 [18th Legislature, Report of the Assembly, Session No. 333 of Thursday, 30 April, 2020],” our translation, available at https://www.camera.it/leg18/410?idSeduta=0333&tipo=stenografico (last accessed on April 1, 2020).

28 Our main concern here is still with rights to health and not with access to healthcare. We will turn more explicitly to access to healthcare in our factual application.

29 https://www.systemicpeace.org/polityproject.html (last accessed July 20, 2021).

30 https://www.cato.org/human-freedom-index-new (last accessed January 15, 2021).

31 https://data.worldbank.org/ (last accessed August 15, 2020).

32 See Daniels (2007) on the right to access healthcare in the context of Rawls.

33 https://www.nytimes.com/article/brazil-coronavirus-cases.html (last accessed September 25, 2021).

34 https://www.bbc.com/news/world-europe-56412790 (last accessed September 25, 2021), https://www.theguardian.com/world/2020/dec/31/covid-control-lessons-from-nicaragua-and-the-isle-of-man (last accessed September 25, 2021), and https://www.nature.com/articles/d41586-021-01714-4 (last accessed September 25, 2021).

35 https://www.theregreview.org/2020/06/10/feldman-japan-lenient-lockdown-conquer-coronavirus/ (last accessed September 25, 2021), https://voxeu.org/article/sweden-s-constitution-decides-its-exceptional-covid-19-policy (last accessed September 25, 2021), and https://www.cbsnews.com/news/national-lockdown-quarantine-president-powers/ (last accessed September 25, 2021),

36 See for example Gates (2015).
